# Time Restricted Feeding to the Light Cycle Dissociates Canonical Circadian Clocks and Physiological Rhythms in Heart Rate

**DOI:** 10.3389/fphar.2022.910195

**Published:** 2022-05-12

**Authors:** Elizabeth A. Schroder, Brian P. Delisle

**Affiliations:** ^1^ Department of Physiology, University of Kentucky, Lexington, KY, United States; ^2^ Department of Internal Medicine, Division of Pulmonary, Critical Care and Sleep Medicine, University of Kentucky, Lexington, KY, United States

**Keywords:** circadian rhythm, heart, homeostasis, feeding, light cycle, heart rate

## Abstract

Circadian rhythms are approximate 24-h biological cycles that optimize molecular and physiological functions to predictable daily environmental changes in order to maintain internal and organismal homeostasis. Environmental stimuli (light, feeding, activity) capable of altering the phase of molecular rhythms are important tools employed by circadian biologists to increase understanding of the synchronization of circadian rhythms to the environment and to each other within multicellular systems. The central circadian clock, located in the suprachiasmatic nucleus (SCN) of the hypothalamus is largely responsive to light and is thought to entrain the phase of peripheral clocks via neurohumoral signals. Mice are nocturnal and consume most of their food during the dark cycle. Early studies demonstrated that altered metabolic cues in the form of time restricted feeding, specifically, feeding mice during the light cycle, resulted in an uncoupling of molecular clocks in peripheral tissues with those from the SCN. These studies showed as much as a 12-h shift in gene expression in some peripheral tissues but not others. The shifts occurred without corresponding changes in the central clock in the brain. More recent studies have demonstrated that changes in cardiac physiology (heart rate, MAP) in response to time of food intake occur independent of the cardiac molecular clock. Understanding differences in the physiology/function and gene expression in other organs both independently and in relation to the heart in response to altered feeding will be important in dissecting the roles of the various clocks throughout the body, as well as, understanding their links to cardiovascular pathology.

## Introduction

Homeostasis is classically defined as the maintenance of internal stability by the action and interaction of complex physiologic mechanisms. Central to the concept of homeostasis are negative feedback mechanisms that continually compensate for challenges to homeostasis. Negative feedback is responsible for maintaining homeostatically regulated variables within a relatively constant range (Richardson et al., 1998). Organisms across the biological spectra, from single cell bacteria to multicellular/multiorgan plants and animals, have evolved reflexive or reactive homeostatic mechanisms that work to respond to sudden behavioral or environmental homeostatic challenges. Organisms have also evolved anticipatory homeostatic mechanisms that alter the regulation of negative feedback mechanisms to prepare the organism for homeostatic challenges caused by predictable changes in the environment and behavior (Moore-Ede, 1986). Circadian rhythms are an example of an anticipatory homeostatic mechanism that enhance internal/organismal homeostatic capacities to predictable homeostatic challenges caused by 24-h rhythms in the environment and behavior. In this review, we use the framework of circadian homeostasis to understand how daily rhythms in behavior and the environment impact circadian mechanisms of homeostasis from the cellular/tissue to the whole organism level.

Circadian biologists work to understand how circadian homeostatic mechanisms promote a physiological advantage. Human lifestyles, which include high intensity light at night, shift work, travel across time zones, night time eating, etc. disrupt predictive behavioral and environmental entrainment cues that are critical for normal circadian homeostatic mechanisms (Bray et al., 2013). The disruption between behavior and environmental cycles has been linked to cancers, obesity, metabolic disease, and cardiovascular disease (West et al., 2017; Kelly et al., 2018; Maury, 2019; Resuehr et al., 2019; Shi et al., 2019; Delisle et al., 2021). Understanding the underlying mechanisms that disrupt circadian homeostatic mechanisms at the cellular and whole organism level will provide insight into how human lifestyles impact health and disease. Basic science studies in rats and mice show that feeding behavior in reference to the light cycle is central to the alignment of circadian homeostatic mechanisms at the cellular/tissue and whole organism level (Damiola et al., 2000; Bray et al., 2013; Schroder et al., 2014; Xin et al., 2021). These animal studies are exciting because they suggest that modifying feeding behavior might represent a powerful therapeutic tool to improve homeostasis and mitigate disease risks in people. In this review, we present the latest findings demonstrating how feeding behavior drives the timing of circadian homeostatic mechanisms with a focus on the heart. Importantly, we discuss how inverting normal feeding behavior leads to significant misalignment between canonical cellular circadian mechanisms in several tissues and physiological circadian rhythms central to homeostasis.

### Time of Day Restricted Feeding and Molecular Rhythms

**FIGURE 1:**
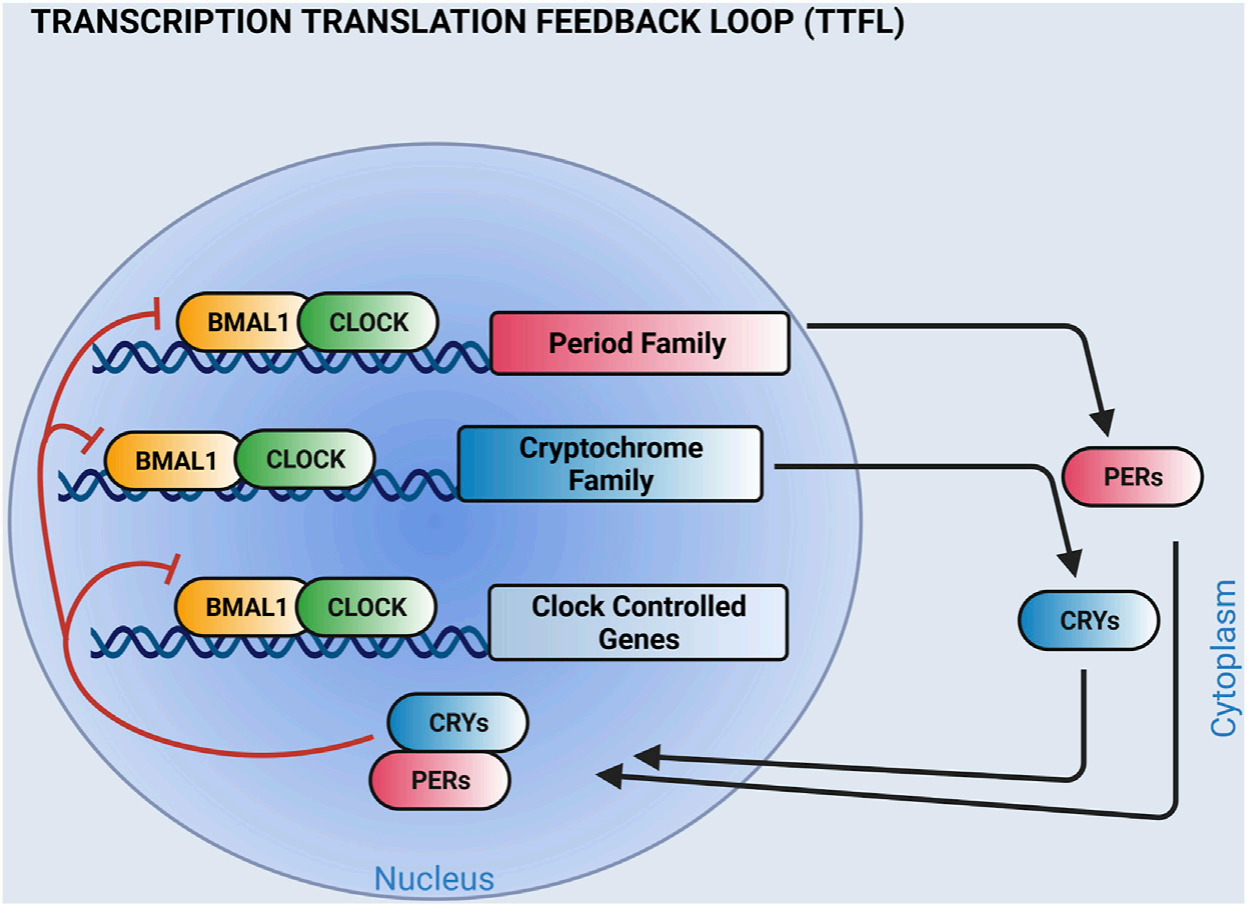
The molecular clock is a transcription translation feedback loop. Shown is a simplified cartoon of the circadian clock mechanism. BMAL1 and CLOCK transcription factor heterodimers bind E-boxes on the promoters of Period (PER 1,2, 3) and Cryptochrome (CRY 1, 2) family members to activate transcription. PER and CRY proteins dimerize and negatively feedback on BMAL1 and CLOCK. The circadian clock mechanism also regulates the tissue-specific expression of clock-controlled genes. Additional feedback loops that contribute to the circadian clock mechanism are not shown. (Created with BioRender.com).

Circadian clocks serve a ubiquitous cellular timekeeping function that regulate the timing of physiological rhythms important for homeostasis. The canonical circadian clock mechanism expressed in most cells is a transcription translation feedback loop that cycles with a periodicity of about 24 h. In mammals, it is formed by four families of core clock genes [*Bmal1*, *Clock*, *Per* (*Period*), and *Cry* (*Cryptochrome*)] (Shearman et al., 2000; Mohawk et al., 2012; Buhr and Takahashi, 2013). BMAL1 and CLOCK are basic helix-loop-helix transcription factors that dimerize and initiate the transcription of the *Per* and *Cry* genes. Translated PER and CRY proteins repress *BMAL1:CLOCK* transactivation, and in so doing, decrease their own expression (Figure 1). As PER and CRY proteins are degraded, the cycle repeats. Components of the circadian clock also regulate the tissue-specific expression of genes outside the cellular timekeeping function and these genes, termed clock-controlled genes, are important for normal physiology (Panda et al., 2002a; Storch et al., 2002; Kornmann et al., 2007; Jeyaraj et al., 2012; Schroder et al., 2013). The importance of this molecular time keeping mechanism cannot be understated, because it is postulated to improve homeostasis by priming effector mechanisms prior to predictable behavioral and environmental challenges.

The location of the central clock in the suprachiasmatic nucleus (SCN) of the hypothalamus was first identified following surgical ablation of the SCN which resulted in arrhythmic behavior patterns (Stephan and Zucker, 1972). Light is the best characterized environmental time cue (zeitgeber), and light entrains the canonical circadian clock in the SCN. Light is transmitted to the SCN via the retinohypothalamic tract from the retina (Gooley et al., 2001; Panda et al., 2002b). The light entrained SCN synchronizes the clocks in peripheral tissues via neurohumoral cues, which in turn align the timing of the circadian clocks in the peripheral tissues to the light cycle. Mice are active and consume ∼80–90% of their calories during the dark cycle (Bray et al., 2013). It is now known that, in addition to light, the timing of feeding behavior can function to align or misalign canonical circadian clocks in the SCN and peripheral tissues (Damiola et al., 2000; Vollmers et al., 2009; Bray et al., 2013; Izumo et al., 2014; Schroder et al., 2014; Schroder et al., 2021; Xin et al., 2021; Zhang et al., 2021).

**FIGURE 2:**
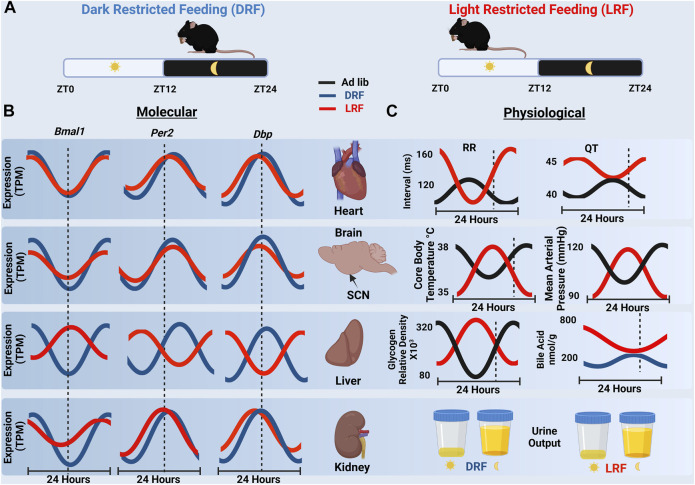
**(A)** Schematic representation of nighttime restricted feeding (DRF), left, and daytime restricted feeding (LRF), right. **(B)** A cartoon representation of RNA-seq expression data of *Bmal1*, *Per2* and *Dbp* over 24 h in the heart, SCN, liver and kidney from mice undergoing DRF and LRF. The dashed line represents ZT12 which is the onset of dark cycle. Notice the ∼12-h shift in gene expression in the liver which is not present in the heart, SCN, or kidney. (TPM, transcripts per million) (Xin et al., 2021) **(C)** Organ specific physiological rhythms in response to ad libitum feeding, LRF or DRF are shown. RR and QT intervals rapidly shift in response to LRF (Schroder et al., 2014). Similar shifts were observed with core body temperature (10) and MAP (27) which are regulated by the SCN under ad libitum conditions. Liver rhythms in liver glycogen content (Pérez-Mendoza et al., 2014) and bile acid production (Ma et al., 2009) also shifted in response to LRF. With the exception of urine output (Zhang et al., 2021) all physiological rhythms shown shift by ∼12 h in response to LRF. (Created with BioRender.com).

Time of day restricted feeding studies that restrict feeding behavior to the light (LRF) or dark (DRF) cycle demonstrate that restricting feeding behavior is a powerful technique to interrogate the interconnections between cellular circadian clocks, physiological circadian rhythms, metabolism, and the time of food (nutrient) intake. Restricted feeding studies that limit food availability to a certain time of day, for a certain period of time, have demonstrated that peripheral clocks respond to time of feeding with changes at both the molecular and physiological level to preserve homeostasis. DRF improved the phase alignment in rhythmic mRNA expression for *Bmal1*, *Per2*, and *Dbp* in the liver, kidney, and heart (Xin et al., 2021). (Figure 2) LRF, feeding mice when they are normally inactive, did not alter the phase of circadian clock signaling in the SCN, but LRF resulted in the misalignment and disruption in the rhythmic expression of clock genes in some (but not all) peripheral tissues (Bray et al., 2013; Xin et al., 2021). Compared to DRF, the phase in the rhythmic mRNA expression for *Bmal1*, *Per2*, and *Dbp* was only modestly impacted by LRF in heart or kidneys, but the phase of the rhythmic expression of *Bmal1*, *Per2*, and *Dbp* mRNA transcripts in the liver shifted by 10–12 h. Xin et al. is one of the few studies to examining sex as a variable in tissue specific gene expression. They found that although the liver clock and adipose clock maintained similar responses to inverted feeding between male and female mice, the kidney and heart clocks were less robust under LRF in male mice (Xin et al., 2021). So how does LRF cause a loss in the alignment or disruption in expression of peripheral clock genes?

The answer to how LRF results in phase misalignment or a disruption of peripheral clocks genes remains incomplete, but several clues can be found in earlier studies. One of which, employed *Per1* deficient mouse embryonic fibroblasts (MEFs), which have a short period length in culture (assessed by *Per2* gene expression), to examine entrainment in peripheral tissues (Pando et al., 2002). Collagen matrix embedded, *Per1* deficient MEFs were implanted under the skin on the back of mice. After a few days, tissue (kidney, skeletal muscle, and MEF containing disks) was harvested from mice over a time course at 4-h intervals and *Per2* mRNA in the implanted MEFs was shown to match the phase and period length of the host mouse isolated tissues. In addition, it was demonstrated that expression was food entrainable, most strikingly similar to that observed in the liver of the host mouse. More recent studies, employing long-term bioluminescence studies [RT-Biolumicorder (Saini et al., 2013)] in freely moving PER2:luciferase reporter mice, which express luciferase downstream of the *Per2* promoter (Yoo et al., 2004), showed that the SCN was required for maintaining phase alignment between organs under constant conditions (ad libitum feeding and constant darkness). DRF and LRF in this study synchronized the phase of peripheral organs in whole body bioluminescence and activity rhythms of PER2:luciferase reporter mice, in both control and SCN ablated mice, with the phase of these rhythms shifting faster in the SCN ablated mice in response to LRF (Guan et al., 2020; Sinturel et al., 2021). Taken together, the aforementioned studies employing MEF implantation and whole animal bioluminescence suggest a complex regulation of feeding induced phase entrainment both systemically and within individual organs. The impact of feeding on phase likely involves regulatory mechanisms emanating from the SCN to synchronize gene expression between organs, as demonstrated by the bioluminescence studies, as well as, circulating and neurohumoral factors or metabolites, as demonstrated by the MEF implantation studies.

Identifying entrainment and food responsive re-entrainment factors for peripheral tissue gene expression has proven elusive. SCN regulated pathways including neurohumoral signaling (autonomic, glucocorticoids, insulin signaling, etc.) and daily changes in core body temperature have been shown to contribute (Balsalobre et al., 2000; Terazono et al., 2003; Buhr et al., 2010; Fougeray et al., 2022). One possible entrainment factor is glucocorticoid signaling. Glucocorticoid receptors are present in most cells in the body but not the SCN. Balsalobre et al. (2000) demonstrated that intraperitoneal injection of mice with dexamethasone, a glucocorticoid receptor agonist, resulted in phase shifts of *Per1*, *Dbp* and *Reverb* gene expression in peripheral tissues (Balsalobre et al., 2000). Of note, dexamethasone treatment is frequently used in circadian bioluminescence cell culture experiments to align molecular clocks (Izumo et al., 2006). Around the same time, Le Minh et al., showed that glucocorticoids slow clock gene phase shifts in the liver resulting from LRF, with little impact on the rate of clock gene phase resetting with a return to ad libitum conditions utilizing adrenalectomized and sham operated mice (Le Minh et al., 2001). These data suggested that glucocorticoid receptor signaling may serve to buffer the peripheral clock against phase shifts in response to transient alterations in feeding patterns and that differential expression/sensitivity of these receptors in tissues throughout the body may mediate changes in gene expression (Quatrini and Ugolini, 2021). Glucocorticoid receptor signaling may play a role in the apparent slow phase shifts observed in the circadian gene expression of peripheral tissues in response to LRF (7–10 days, liver) (Damiola et al., 2000). Another study demonstrated that circadian gene expression in the liver requires a functional insulin receptor to entrain the circadian clock mechanism to LRF (Fougeray et al., 2022). These data suggest an important role for insulin signaling/hepatocyte insulin sensitivity in regulating the liver circadian clock with important implications for maintaining blood glucose homeostasis, as well as how circadian disruption could effect metabolic homeostasis and contribute to metabolic disorders (e.g., metabolic disease, obesity and diabetes) (Fougeray et al., 2022). Future studies may identify tissue specific signaling mechanisms that regulate the circadian clock to alterations in the timing of feeding.

### Time of Day Restricted Feeding and Circadian Rhythms: Canonical Circadian Clocks Not Required

Mouse models that are currently available to dissect the role of the molecular clock at the systems level and within individual tissues, suggest a direct link between the canonical circadian clock mechanism in the SCN, circadian clocks throughout the body, and circadian homeostasis of several homeostatically regulated physiological variables including mean arterial pressure (MAP). Studies utilizing SCN ablation, germline *Bmal1* knock out mice (*Bmal1* ^−/−^), or conditional systemic *Bmal1* ^*−/−*^ knock out mice show that these animals lose the circadian rhythm in MAP and HR (heart rate) (Yang et al., 2016).

MAP is normally homeostatically maintained within a narrowly defined range so organs and tissues can be perfused with blood to meet their metabolic demands (De Hert, 2012). The maintenance of MAP within constant range occurs by numerous nested negative feedback mechanisms that regulate cardiac output, total peripheral resistance, and blood volume (Nishida et al., 2012; Raven and Chapleau, 2014; Dampney, 2016). Central to the homeostatic regulation of MAP is heart rate and cardiac output (Richardson et al., 1998; De Hert, 2012). The importance of the heart as an effector in the homeostatic regulation of MAP is underscored by the fact that the loss of heart function causes sudden cardiac death, the worldwide leading cause of death (Srinivasan and Schilling, 2018). The autonomic nervous system dynamically regulates heart function on a beat-to-beat basis. Environmental and behavioral homeostatic challenges cause efferent sympathetic and parasympathetic-induced signaling to change cardiac output primarily through the modulation of HR. Like many other critically important homeostatic effector mechanisms, the autonomic nervous system not only changes HR in response to homeostatic challenges, but also in anticipation of daily environmental and behavioral cycles (Joyner and Limberg, 2014; Raven and Chapleau, 2014). It has been proposed that circadian homeostatic mechanisms prime effectors to improve their responsivity to predictable environmental and behavioral challenges. Normally basal HR increases in anticipation of the active cycle and decreases prior to the rest cycle. In the next section we discuss surprising new data that suggest the canonical circadian clocks in the SCN (i.e. the central clock) or heart do not appear to be directly responsible for priming the heart to generate these anticipatory rhythms in HR. Studies using LRF are now beginning to point to a role of another oscillator, perhaps the food entertainable oscillator (FEO), as being a driver in generating the circadian rhythm in HR in mice (Zhang et al., 2021).

### Eat Your Heart Out Canonical Clocks

Lesioning the SCN (central clock) in the brain under ad libitum conditions results in a loss of circadian rhythm in HR in rats and mice (Sano et al., 1995; Scheer et al., 2001; Tong et al., 2013). A similar loss in the 24-h rhythm in HR is seen in mice undergoing both beta-adrenergic and muscarinic receptor blockade. These studies, along with the identification of neuronal pathways linking the SCN to autonomic nuclei, suggested that the circadian clock mechanism in the SCN modifies autonomic regulation to generate the circadian rhythm in HR. If true, then germline deletion of *Bmal1* should also result in the loss of the circadian rhythm in the HR. Indeed, *Bmal1* ^−/−^ mice show a loss in the 24-h rhythms in sympathoadrenal function and HR (Lefta et al., 2012). The loss in the 24-h rhythm in HR was also seen in the inducible systemic deletion of *Bmal1* in adult mice (Yang et al., 2016). To better understand how the canonical circadian clock in the heart may impact 24-h rhythms in HR, mouse models that disrupt the circadian clock mechanism by knocking out *Bmal1* in a cardiac specific manner were developed (Schroder et al., 2013; Young et al., 2014; Yang et al., 2016; Zhang et al., 2021). Mouse models in which *Bmal1* is deleted from the heart from birth or induced in adult cardiomyocytes showed a circadian and 24-h rhythm in HR. Taken together the data suggest the SCN and the canonical circadian clock mechanism outside the heart regulate autonomic activity to generate the daily rhythm in HR.

The model that the canonical clock in the SCN modifies autonomic signaling to generate the 24-h rhythm in HR was challenged when it was demonstrated that LRF shifted the phase in the 24-h rhythm of HR by ∼15 h but not the circadian clock mechanism in the SCN (Schroder et al., 2014). The shift in the 24-h rhythm in HR did not appear to be secondary to changes in the circadian clock in the heart, because LRF only caused a modest shift in the phase of the canonical circadian clock in the heart (Figure 2). Additional studies confirmed that the impact the LRF had on the phase of the 24-h rhythm in HR was not dependent on the canonical circadian clock mechanism in the heart, because mice in which the deletion of *Bmal1* was induced in adult cardiomyocytes undergoing LRF showed a large phase shift in the 24-h rhythm of the HR similar to control mice (Schroder et al., 2021). Perhaps even more surprising were studies utilizing germline *Bmal1* ^*−/−*^ mice, which showed that the loss in the 24-h rhythm in HR was restored in mice undergoing LRF (Zhang et al., 2021). Together, the data now suggest a model, whereby, the canonical circadian clock mechanism is not needed to generate a 24-h rhythm in HR. The loss of the 24-h rhythm in HR after the ablation of the SCN or in the germline or inducible *Bmal1* knock out mice may be attributed to arrhythmic feeding behaviors. The data suggest that the loss in the time of day feeding behavior in these mouse models might underlie the loss or damping of circadian rhythms in HR. There are several possibilities that might explain the dissociation between the phase in the 24-h rhythms of the canonical circadian clocks in the SCN, the heart, and other peripheral tissues and HR. One candidate is FEO. Consistent with the FEO contributing to the circadian rhythm in HR during LRF is that the circadian rhythm in HR aligns with time of feeding even in mice housed in constant darkness (free running conditions) (Zhang et al., 2021).

Importantly, the LRF-induced dissociation between canonical circadian clock mechanisms in tissues and homeostatically regulated effector mechanisms and variables is not limited to the HR (Figure 2). There are several examples in mice undergoing LRF where phase of the 24-h rhythms in canonical circadian clocks in peripheral tissues stays closely aligned to the light cycle, but physiological rhythms align to feeding behavior. Examples include the MAP, core body temperature, liver glycogen content, and bile acid levels (but not urine output) (Figure 2). Future studies are needed to determine the impact that the FEO as well as the dissociation between canonical circadian clock signaling and these physiological rhythms have on overall homeostasis and health.

## Discussion

Many studies have demonstrated the presence of the FEO, but the anatomical location and molecular underpinnings of the FEO are not yet known and understanding the role of FEO beyond food anticipatory activity is limited (Mendoza, 2007). Any discussion of HR being under the control of FEO is absent from the literature. In addition, genes responsible for the canonical molecular clock do not seem to impact FEO, as it remains intact in most circadian clock gene knockout models (Pendergast and Yamazaki, 2018).

Rodent models have demonstrated that feeding behavior in reference to the light cycle is central to the alignment of circadian homeostatic mechanisms and that LRF may cause misalignment between peripheral tissue molecular clocks and physiological rhythms important for systemic homeostasis. Components of this misalignment are likely present in a recent study examining a model of shift work in rats. They demonstrated that a restoration of circadian gene expression, with a lower amplitude, in the heart when food was restricted to non “work” hours was not sufficient to prevent significant cardiac fibrosis (Trott et al., 2022). Although a complete understanding of the impacts of feeding on homeostatic mechanisms at the molecular, cellular, tissue, organ and organismal level is currently unavailable, new scientific tools and animal models are beginning to offer some clarity.

Why don’t molecular rhythms phase shift to the same degree in all tissues in response to LRF? And why do phase shifts occur on a longer time scale (days to weeks), for instance genes in the heart and kidney do not shift very much while those in the liver shift ∼12 h after a week of LRF (Xin et al., 2021)? While the mechanisms responsible for these changes in gene expression may not yet be known perhaps this may reflect tissue specific roles of the molecular clock. For example, the phase of the circadian clock in the liver in mice shifts to align with feeding behavior in LRF mice. The shift in the liver circadian clock relies on the presence of hepatic insulin receptors (Fougeray et al., 2022). Tissues that have a limited phase shift like the heart and kidney are continuously functioning and gene expression may not shift as much as a result while the liver is highly metabolic and responsive to the presence of food, functioning to homeostatically regulate blood glucose, and may shift gene expression for appropriate food utilization. Peripheral tissues are subject to distinct homeostatic mechanisms and respond to different circulating metabolites and hormones secreted in response to feeding. For instance, the liver clock may shift in response to LRF because the liver is an effector for blood glucose, which is homeostatically regulated, to alter storage and release. This shift may initially be a reactive homeostatic response to the new time of feeding and then progress to an anticipatory homeostatic mechanism with prolonged restricted feeding.

In this review we focused primarily on HR because it is critical for cardiac output and loss of cardiac output results in sudden cardiac death. Since the 24-h rhythm in HR strongly depends on autonomic signaling, it might be that the FEO regulates the HR via the autonomic signaling. This could explain why LRF causes similar shifts in the phase of other physiological rhythms known to be impacted by the autonomic nervous system (e.g., MAP and body temperature). However, because MAP and core temperature are homeostatically regulated variables, their regulation is much more complex than HR. For example, MAP depends on the diameter of the arteries and veins, which impacts total peripheral resistance and blood distribution between the arteries and veins, as well as blood volume, which reflects the balance between fluid intake and fluid loss (passively or by the kidney).

Although the idea that FEO might drive the 24-h rhythm in HR is exciting, there is evidence that this might not be the case in all animal models. For example, recent LRF studies in rat show a very different type of dissociation between the canonical circadian clock in the heart and HR. Unlike mice, the phase in the canonical circadian clock in the heart realigns with feeding behavior (similar to the liver), but LRF for 5-days has less of an effect on the phase of the 24-h rhythm of HR than mice (Wu et al., 2010). Regardless, both mouse and rat animal models show LRF can cause dissociation between the phases of canonical circadian clocks in peripheral tissues and physiological rhythms. Understanding the mechanisms for this in different model systems will be important for future studies on reactive and circadian homeostasis.

The data strongly suggest that canonical circadian clock rhythms and physiological rhythms are regulated by distinct oscillator mechanisms. Lifestyles and disease that disrupt and misalign circadian and physiological rhythms have been shown to increase cardiovascular risk in humans (Scheer et al., 1999; Scheer et al., 2009; Chellappa et al., 2021). Understanding the impact that time of feeding has on cardiovascular homeostasis in animal models and humans will enable the development of novel targeted approaches to mitigate cardiovascular disease risk.
